# Missense Mutation Lys18Asn in Dystrophin that Triggers X-Linked Dilated Cardiomyopathy Decreases Protein Stability, Increases Protein Unfolding, and Perturbs Protein Structure, but Does Not Affect Protein Function

**DOI:** 10.1371/journal.pone.0110439

**Published:** 2014-10-23

**Authors:** Surinder M. Singh, Swati Bandi, Dinen D. Shah, Geoffrey Armstrong, Krishna M. G. Mallela

**Affiliations:** 1 Department of Pharmaceutical Sciences, Skaggs School of Pharmacy and Pharmaceutical Sciences, University of Colorado Anschutz Medical Campus, Aurora, Colorado, United States of America; 2 Department of Chemistry and Biochemistry, University of Colorado Boulder, Boulder, Colorado, United States of America; 3 Program in Structural Biology and Biochemistry, University of Colorado Anschutz Medical Campus, Aurora, Colorado, United States of America; Southern Illinois University School of Medicine, United States of America

## Abstract

Genetic mutations in a vital muscle protein dystrophin trigger X-linked dilated cardiomyopathy (XLDCM). However, disease mechanisms at the fundamental protein level are not understood. Such molecular knowledge is essential for developing therapies for XLDCM. Our main objective is to understand the effect of disease-causing mutations on the structure and function of dystrophin. This study is on a missense mutation K18N. The K18N mutation occurs in the N-terminal actin binding domain (N-ABD). We created and expressed the wild-type (WT) N-ABD and its K18N mutant, and purified to homogeneity. Reversible folding experiments demonstrated that both mutant and WT did not aggregate upon refolding. Mutation did not affect the protein's overall secondary structure, as indicated by no changes in circular dichroism of the protein. However, the mutant is thermodynamically less stable than the WT (denaturant melts), and unfolds faster than the WT (stopped-flow kinetics). Despite having global secondary structure similar to that of the WT, mutant showed significant local structural changes at many amino acids when compared with the WT (heteronuclear NMR experiments). These structural changes indicate that the effect of mutation is propagated over long distances in the protein structure. Contrary to these structural and stability changes, the mutant had no significant effect on the actin-binding function as evident from co-sedimentation and depolymerization assays. These results summarize that the K18N mutation decreases thermodynamic stability, accelerates unfolding, perturbs protein structure, but does not affect the function. Therefore, K18N is a stability defect rather than a functional defect. Decrease in stability and increase in unfolding decrease the net population of dystrophin molecules available for function, which might trigger XLDCM. Consistently, XLDCM patients have decreased levels of dystrophin in cardiac muscle.

## Introduction

**Figure 1:**
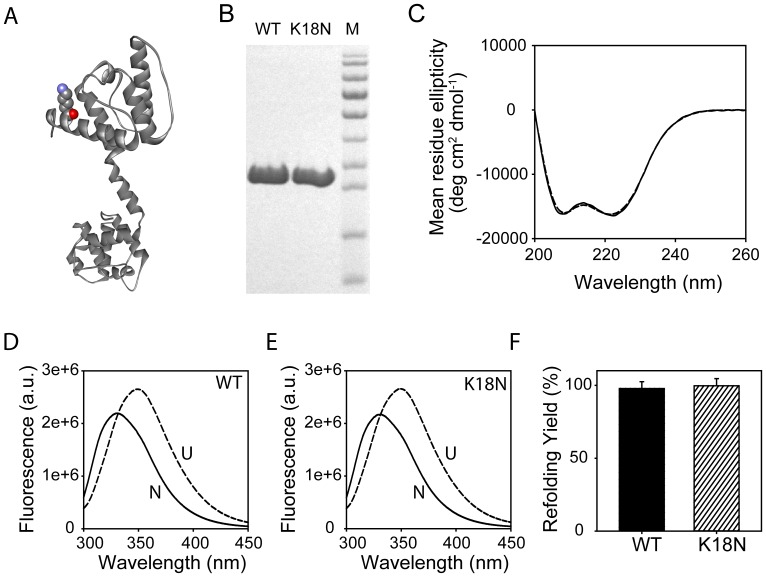
Expression, purification, and biophysical characterization of the K18N mutant and the WT. (A) Ribbon diagram of the X-ray crystal structure of dystrophin N-ABD (1DXX.pdb) with the mutated lysine shown in a space-filling or cpk (Corey, Pauling, and Koltun) model. (B) SDS-PAGE of purified proteins. Lane M corresponds to molecular weight markers (bottom to top: 11, 17, 26, 34, 43, 56, 72, 96, 130, and 170 kDa respectively). (C) CD spectra of the WT (solid line) and the mutant (dashed line). (D) Fluorescence spectra of the native (solid line) and denatured (dashed line) WT. (E) Fluorescence spectra of the native (solid line) and denatured (dashed line) mutant. (F) Refolding yields of the two proteins.

X-linked dilated cardiomyopathy (XLDCM) involves progressive heart muscle degeneration and is a lethal disorder leading to death at an early age in male patients [1]. No cure is currently available for XLDCM. The only viable option for treating XLDCM is heart transplant. Mutations in the gene coding for a vital muscle protein dystrophin trigger XLDCM [1]–[12]. Although dystrophin mutations are in general linked to Duchenne/Becker muscular dystrophy (DMD/BMD) [13], no apparent sign of skeletal muscle degeneration were observed in XLDCM patients [1], which distinguishes them from DMD/BMD patients. The major function of dystrophin is to link intracellular cytoskeleton with the extracellular matrix [14]. This tethering helps in maintaining the integrity of the myocardial membrane against continuous cycles of stretches and contractions, and plays an important role in cell adhesion [15], [16]. Disease-causing mutations in dystrophin include premature stop codons, frameshift mutations, and missense mutations. Premature stop codons and frameshift mutations express partial or incorrect protein, and hence trigger the disease because of the lack of a functional protein. However, how missense mutations trigger the disease is not clear, in which the expressed protein is of similar length to that of the wild-type protein. Our goal is to understand the disease mechanisms at the fundamental protein level at an atomic resolution. Such molecular knowledge will help in developing effective therapies to treat XLDCM, for example, small molecule therapeutics and compensatory gene constructs. For this purpose, we need to first understand the effect of disease-causing mutations on the structure and function of dystrophin. In this work, we examined the case of a missense mutation where lysine at the 18^th^ position in the dystrophin amino acid sequence is replaced by asparagine (Lys18Asn, or K18N). This mutation occurs in the N-terminal actin-binding domain (N-ABD) of dystrophin (Fig. 1A). The K18N mutation is associated with the hallmarks of XLDCM (LVEDD 70 mm, shortening fraction 8%, ejection fraction 19%, and large ventricular thrombus). Among 141 control patients examined, none harbored the K18N mutation indicating its 100% linkage with XLDCM [17]. Creatine kinase levels were 11,300, which were as high as observed in more severe DMD patients; however, no sign of skeletal myopathy were recorded, confirming that the K18N mutation is a XLDCM case. Here, we analyzed the effect of mutation on dystrophin structure and function using various biophysical and structural methods. Our results indicate that the K18N mutation decreases thermodynamic stability, accelerates unfolding, perturbs protein structure, but does not affect protein function.

## Materials and Methods

### Expression and purification

Wild type (WT) dystrophin N-ABD (residues 1–246) was cloned into pET28a vector as described previously [18]. The K18N mutant was created using WT as the template using quick mutagenesis protocol (Qiagen), and was confirmed by DNA sequencing. Both WT and mutant proteins were transformed into BL21(DE3) by heat shock, and glycerol stocks were made for further expression and purification. WT and mutant were expressed in 2 L lysogeny broth (LB) media and pellets were used for purification. Cells were lysed with sonication and proteins were purified from the supernatant by nickel affinity chromatography. Pure proteins were dialyzed against PBS (0.1 M sodium phosphate, 0.15 M sodium chloride, pH 7) and used for experiments described below.

### Fluorescence and circular dichroism spectroscopy

Fluorescence spectra of native and unfolded states (in 8 M urea) of WT and mutant proteins (1 µM) in PBS buffer were recorded by exciting the samples at 280 nm (QuantaMaster, PTI). CD spectra (5 µM protein in PBS) were recorded on an Applied Photophysics ChirascanPlus spectrometer. Mean residue ellipticity (MRE) of the proteins were calculated from the CD values in millidegrees using the equation [19], [θ] = CD in millidegrees/(path length in millimeters x molar concentration of protein x number of residue).

### Thermal melts

WT and mutant proteins (1 µM each in PBS) were used for calculating the T_m_ (midpoint of thermal denaturation transition) by using CD signal at 222 nm with increasing temperature in a continuous ramp mode (1°C/min). Normalized signal was plotted against temperature, and the data were fitted to a two-state equilibrium unfolding model in SigmaPlot as described earlier [18].

### Chemical denaturation melts

WT and mutant proteins (1 µM each in PBS) were subjected to equilibrium denaturation melts. Urea was used as the denaturant. Protein solutions at varying urea concentrations were prepared and equilibrated for 1 hr. CD signal at 222 nm was measured (ChirascanPlus, Applied Photophysics) for all samples. Normalized signal was plotted against increasing urea concentration. Free energy of unfolding, ΔG, was determined by fitting the curve to a two-state unfolding model [20], [21].

For fluorescence urea denaturation melts, samples were prepared using the same method as described above. Samples were excited at 280 nm and the change in emission intensity at 350 nm was monitored (QuantaMaster, PTI). Normalized fluorescence signal was fitted to a two-state unfolding model using SigmaPlot.

### Structural analysis by NMR

For labeling WT and mutant proteins with ^15^N, 2 L LB culture (optical density ∼2) pellet was resuspended into 1 L sterile M9 media (48 mM Na_2_HPO_4_, 22 mM KH_2_PO_4_, 8.5 mM NaCl, 18.6 mM ^15^NH_4_Cl, 2 mM MgSO_4_, 0.1 mM CaCl_2_, 0.4% glucose), and was grown for 2 hrs at 310 K. Protein expression was induced with 0.5 mM isopropyl β-D-1-thiogalactopyranoside (IPTG) overnight, and the labeled proteins were purified to high homogeneity using protocols described above for unlabeled proteins. Proteins were concentrated using Centricon (Amicon Ultra, Millipore) to 150 µM and standard HSQC-TROSY NMR spectra were recorded on a 900 MHz Varian NMR instrument. Spectra were processed using NMRPipe [22]. A peak table was generated with arbitrary index number for WT protein as described earlier [18]. Same peak index number was used for the peaks of mutant protein to calculate the chemical shift differences. Pictures showing overlay of the spectra were generated with NMRView software.

### Refolding yield

Mutant and WT proteins were solubilized in PBS buffer containing 8 M urea to a final concentration of 10 µM. Molar extinction coefficient (46075 M^−1^cm^−1^ @ 280 nm) used for quantification was calculated using ExPASy program (http://www.expasy.org/). Solubilized proteins in 8 M urea were refolded by 10 times dilution into PBS and incubated for one hr to reach equilibrium. Refolded solutions were centrifuged at 30,000 g for 10 min and proteins in the supernatant were measured using absorbance at 280 nm.

### Stopped flow kinetics

Unfolding kinetics of the mutant and WT proteins were monitored using an Applied Photophysics stopped flow assembly attached to a ChirascanPlus spectrometer. Proteins (10 µM each) were diluted 10 times into PBS buffer containing high denaturant, and changes in the CD signal at 222 nm were recorded. An average of 20 traces was fit to an exponential function using SigmaPlot to determine the rate constant.

### Actin binding affinity

Skeletal muscle G-Actin (Cytoskeleton, Denver, USA) was polymerized (7 µM, final concentration) and incubated at room temperature with varying concentrations of either the WT or the mutant protein. Above mix (final volume 100 µl) was centrifuged at 100,000 g for 30 min (sw55Ti rotor, Beckman Optima LE80K) and pellets were solubilized in 30 µl SDS-PAGE loading buffer. Half of this was boiled and subjected to SDS-PAGE electrophoresis and was stained with coomassie brilliant blue. Intensities of the individual bands were determined using the QuantityOne software on Biorad Gel Doc XR. These intensity values were corrected for the differential staining of proteins with Coomassie Blue as described before [23], [24]. Ratios of the corrected intensities were used to determine the fraction bound of actin and free protein (either the WT or mutant). Fraction bound of F-actin was plotted against free protein to obtain the binding curve.

### Actin depolymerization Assay

G-actin labeled with pyrene (40 µM) was polymerized in polymerization buffer (10 mM Tris, 2 mM MgCl_2_, 50 mM KCl, 1 mM ATP, pH 7.5) and diluted 20 times into G-Actin buffer (5 mM Tris, 0.2 M CaCl_2_, 0.2 mM ATP, pH 8.0) containing either the WT or the mutant protein (16 µM). Change in pyrene excimer fluorescence signal was monitored against time.

## Results

### Protein expression, purification, and biophysical characterization

Both proteins (WT & K18N) were expressed and purified to high homogeneity (Fig. 1B). Circular dichroism (CD) in the far-UV region, typically in the wavelength range 200–260 nm where the peptide group absorbs, is generally used to characterize the secondary structure of proteins [19]. Each secondary structure has distinct maxima and minima in this wavelength range. For the K18N mutant, its far-UV CD spectrum is similar to that of the WT (Fig. 1C), indicating that the mutation did not affect the protein's secondary structure. The minima at 222 nm and 208 nm are characteristic of an α-helical structure [19], which are consistent with the crystal structure of the WT protein (Fig. 1A). In addition, the mutant shows similar fluorescence spectra as that of the WT in both native and unfolded states (Figs. 1D & E). Intrinsic protein fluorescence originates mainly from tryptophan residues [25]. WT protein contains eight tryptophans distributed all across the protein. Tryptophan fluorescence is in general sensitive to the local environment around the aromatic sidechain. Identical fluorescence spectra for the mutant and the WT indicate that there is no effect of mutation on the local environment of the tryptophan residues. The native fluorescence of both the WT and mutant are blue-shifted with respect to their unfolded states. For both proteins, fluorescence emission maxima of the native and unfolded states occur at 330 nm and 349 nm respectively. The blue shift in the tryptophan emission maximum indicates that these residues are located in the native protein interior, not accessible to solvent water [25]. For tryptophans exposed to water, emission maximum occurs at around 355 nm, whereas for tryptophans buried in the hydrophobic protein interior, the emission maximum occurs at around 325 nm. The observed blue shift of 19 nm indicates that at least some of the eight tryptophan residues are significantly buried in the folded protein interior, implying that both proteins are well-folded in solution and the mutation has no effect on the local environment around the tryptophan residues. For both proteins, the fluorescence is increased upon unfolding, which indicates that the tryptophan fluorescence is quenched in the native state by the neighboring amino acids, similar to that observed in other proteins [26], [27].

### The K18N mutant is less stable than the WT

**Figure 2:**
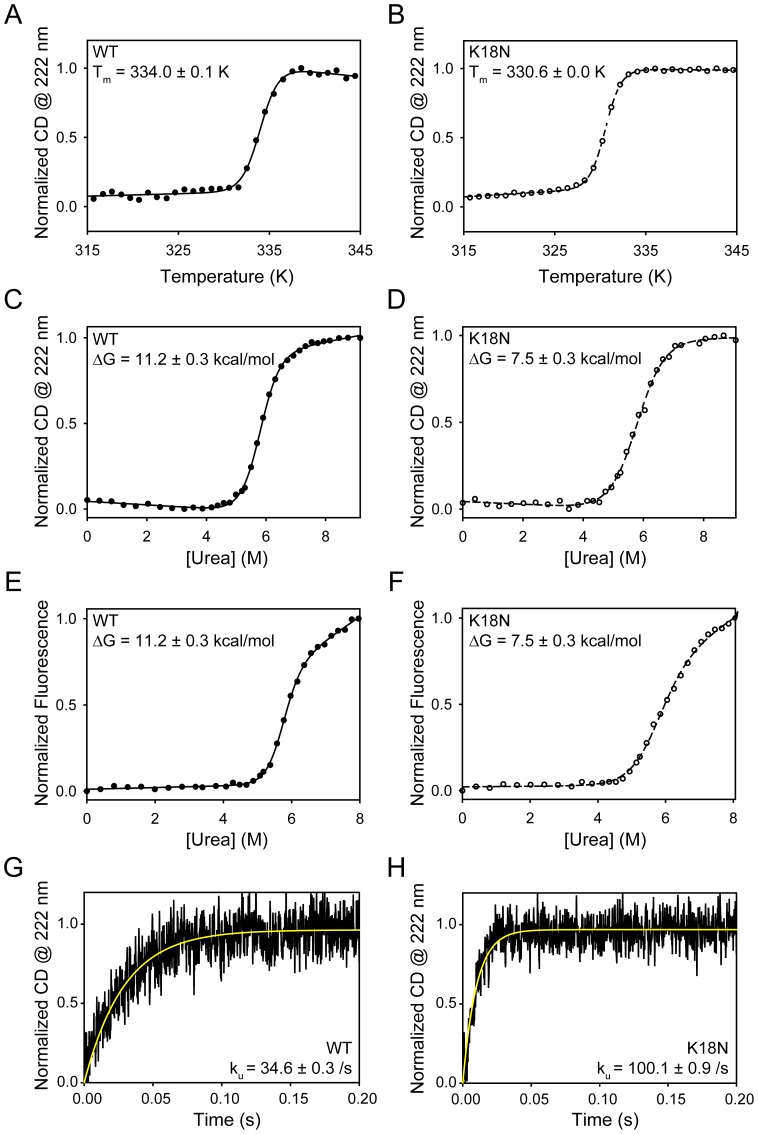
Effect of K18N mutation on protein stability and unfolding. (A) and (B) show the thermal melts of the WT and the mutant respectively. Circular dichroism (CD) @ 222 nm was used to monitor protein unfolding. (C) and (D) show the denaturant melts of the WT and the mutant measured using CD @ 222 nm as the signal. (E) and (F) show the corresponding denaturant melts with fluorescence (λ_ex_ = 280 nm, λ_em_ = 350 nm) as the signal. (G) and (H) show the stopped-flow unfolding kinetics of the WT and the mutant starting from their native states, measured using CD @ 222 nm as the signal.

Thermal and chemical denaturation are commonly used to measure protein stability [18], [28]. In thermal denaturation, solution temperature is increased to destabilize the protein structure, whereas in chemical denaturation, a denaturant such as urea is added to the solution to destabilize the protein structure. Thermal denaturation melts of the WT and the mutant show that both proteins unfold in a sigmoidal fashion (Figs. 2A & B), indicating their cooperative unfolding. Further, these melting curves indicate that the mutant requires lesser temperatures to unfold compared to the WT, indicating its decreased stability. Fitting these curves to a standard sigmoidal function [18] resulted in midpoint transition temperature (T_m_) values of 334.0±0.1 K (or, 61°C) and 330.6±0.0 K (or, 57.6°C) for the WT and the mutant respectively. However, thermal denaturation is irreversible, and denatured proteins could not be renatured back as they resulted in serious aggregation. Hence, these thermal melts can only be used to qualitatively assess the decreased stability of the mutant.

To obtain the true thermodynamic stability difference, we used equilibrium chemical denaturation [29]. We first showed that the folding of both WT and the mutant are completely reversible. Starting from their unfolded states in high denaturant, both proteins refold by 100% (Fig. 1F). No aggregation was observed for both proteins during refolding. Therefore, chemical denaturation can be used to measure thermodynamic protein stability. When the protein denaturation was monitored as a function of increasing urea concentration using either CD or fluorescence as the signal, both the WT and the mutant unfolded in a cooperative, sigmoidal transition. Fitting these denaturant melts to a standard two-state equation [20], [21] resulted in Gibbs free energy of unfolding, ΔG values of 11.2±0.3 kcal/mol and 7.5±0.3 kcal/mol for the WT and the mutant respectively. These values indicate that the K18N mutant is thermodynamically less stable than the WT by 3.7±0.4 kcal/mol. This stability difference can be understood as follows. The non-functional unfolded state population is given by [Unfolded]  =  [Native] exp (−ΔG/RT), where R is the gas constant, and T is absolute temperature in kelvin. That means, the decrease in ΔG by 3.7 kcal/mol increases the unfolded state population by 529 times in the case of mutant when compared to the WT.

### The K18N mutant unfolds faster than the WT

Millisecond stopped-flow methods were used to monitor unfolding of the two proteins [30]. Starting from their native states with no denaturant, unfolding was initiated by mixing with high denaturant. The mutant unfolds three times faster than the WT (Figs. 2G & H). Unfolding rate constants for the mutant and the WT were 100.1±0.9/s and 34.6±0.3/s, respectively. These experiments along with the above chemical denaturation melts indicate that the mutant has lower kinetic and thermodynamic stability compared to the WT protein.

### The K18N mutant shows structural differences at numerous amino acids

**Figure 3:**
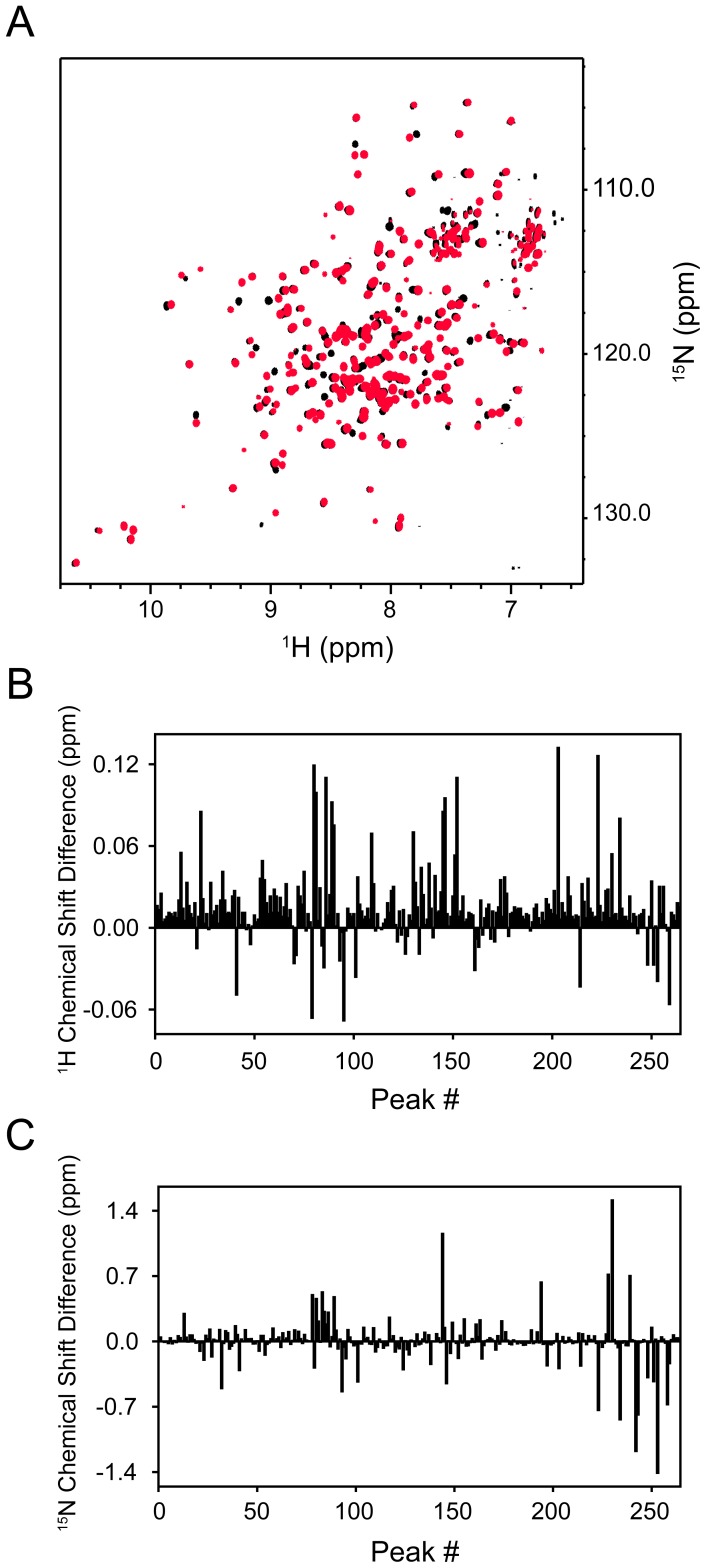
Effect of K18N mutation on protein structure. (A) Overlay of 2D ^15^N-^1^H TROSY-HSQC spectra of the WT (black) and the mutant (red). Each cross-peak represents a unique residue in the protein. (B) and (C) show the change in the ^1^H and ^15^N chemical shifts of the mutant with respect to the WT.

Two-dimensional NMR experiments were used to monitor the amino acid residue-resolved changes in protein structure [18]. Fig. 3A shows the overlay of heteronuclear ^1^H-^15^N HSQC spectra of the mutant and the WT. Each cross-peak corresponds to one amide in the protein, which is mainly the mainchain amide coming from an individual amino acid. Any change in the cross-peak position represents a local structural change at that particular amino acid, which in general may not be detectable by global probes such as circular dichroism. The overall cross-peak pattern of the mutant is similar to that of the WT (Fig. 3A). However, when individual cross-peak positions were compared, we do see clear differences (Figs. 3B & C). Both ^1^H and ^15^N chemical shifts of many residues show significant changes. The fact that NMR chemical shift differences can be seen at several residues implies that the effect of mutation is propagated over long distances in the protein structure. However, the overall secondary and tertiary structures of the protein were not affected by the mutation, as evident from no changes in the CD and fluorescence spectra (Figs. 1D & E).

### The K18N mutant does not affect the actin-binding function

**Figure 4:**
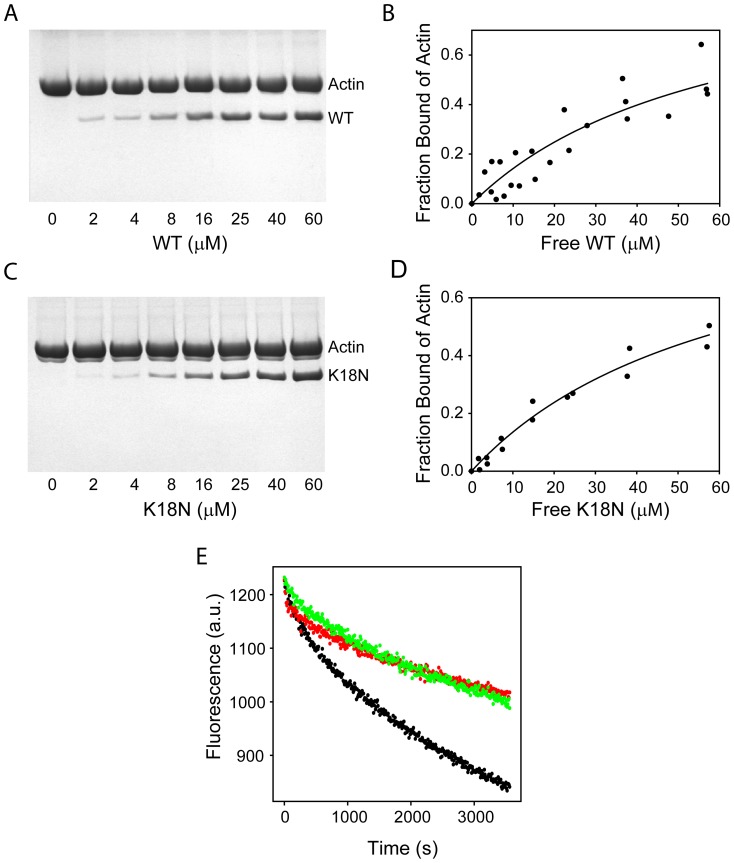
Effect of K18N mutation on protein function. (A) – (D) show the results from actin co-sedimentation assay. (A) & (C) show SDS-PAGE of the pellets after co-sedimentation of varying concentration of the WT or the mutant with a fixed concentration of F-actin. The upper and lower bands in SDS-PAGE indicate actin and bound WT (or mutant) in the pellet. (B) and (D) show the fraction of actin bound as a function of free protein, calculated from the protein band intensities from SDS-PAGE after correcting for the differential staining of the dye. (E) Actin depolymerization assay. Green and red represent the depolymerization kinetics of F-actin in the presence of the WT or the mutant, respectively. Black trace corresponds to the kinetics in the absence of the WT or the mutant.

Traditional actin co-sedimentation assays were used to measure the actin binding affinity of the WT and the mutant [23], [31], [32]. Varying concentrations of the protein (0–60 µM) were added to a solution containing a fixed concentration of F-actin (final concentration of 7 µM) and were centrifuged at high speed (100,000g for 30 min) to separate the free protein from the protein bound to F-actin. The unbound protein remained in the supernatant, whereas F-actin and the bound protein pelleted at the bottom of the tube. The concentration of the bound protein was determined using SDS−PAGE densitometry (Figs. 4A & C), and was corrected using bovine serum albumin (BSA) as a standard to account for the differential staining of coomassie blue to proteins [23], [24]. These corrected values were used to calculate the fraction of actin bound as a function of the free protein concentration (Figs. 4B & D). Actin-binding curves of the mutant and the WT were similar, indicating that the mutation does not affect the actin-binding function. These binding curves were fit to a standard binding equation, and the obtained K_d_ value of ∼45 µM matches the K_d_ value predicted from earlier cryo-EM studies [33].

Dystrophin binding to F-actin offers partial protection against its depolymerization [14]. We examined the effect of K18N mutation on such depolymerization (Fig. 4E). For this experiment, F-actin with individual actin molecules labeled with pyrene fluorophores was diluted into G-actin buffer, and the decrease in pyrene excimer fluorescence was followed. The main principle behind pyrene excimer fluorescence is that when an excited pyrene molecule is close in three-dimensional space to a neighboring, ground-state pyrene, their aromatic rings stack against each other to form a dimer (excited-state dimer or excimer) [34], [35]. This results in a new fluorescence emission band, which is not present in the fluorescence spectrum of monomeric pyrene. When actin molecules labeled with pyrene are close in F-actin, excimer fluorescence will be higher. With depolymerization, excimer fluorescence will decrease, as the actin molecules in F-actin are falling apart into monomers. Pyrene excimer fluorescence decreases at the same rate for both WT and the mutant implying that both proteins protect F-actin to the same extent (Fig. 4E). This assay along with the above co-sedimentation assay indicates that the mutation did not affect the actin-binding function of the protein.

## Discussion

Our results show that the K18N mutation decreases protein stability and perturbs protein structure, but does not affect function. This is in contrary to common presumption that disease-causing mutations always affect protein function. Therefore, K18N mutation can be classified as a protein stability defect, and not as a protein function defect. Decrease in protein stability increases the non-functional unfolded protein population, given by [Unfolded]  =  [Native] exp (−ΔG/RT), where ΔG is the Gibbs free energy of unfolding, R is the gas constant, and T is the absolute temperature in kelvin. Unfolded or improperly folded protein molecules are in general broken into small peptides by the proteasome machinery, and the resultant peptides are used for further protein synthesis [36]. That means, any mutation that decreases the protein stability will result in a depletion of the net functional protein molecules. This might be the disease triggering factor for XLDCM. Consistently, XLDCM patients were reported to have decreased levels of dystrophin in cardiac muscle [11], [12], [17].

**Figure 5:**
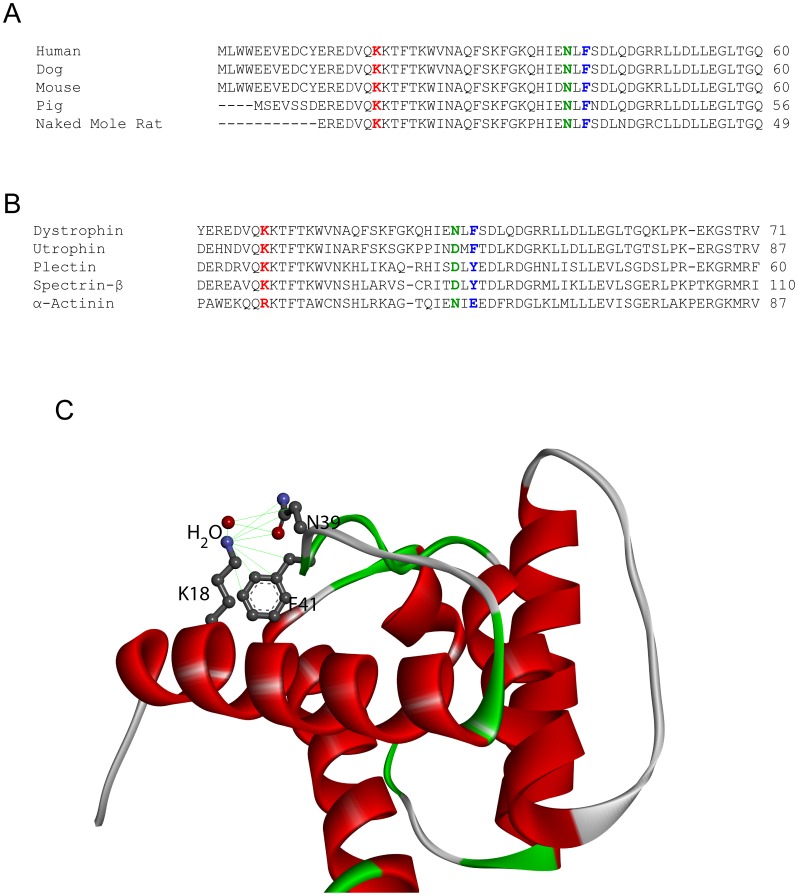
Sequence and structural analysis of the K18N mutation. (A) Sequence alignment of dystrophin from various mammals. Lysine at the 18^th^ position, asparagine at the 39^th^ position, and phenyl alanine at the 41^st^ position are shown in red, green, and blue colors respectively. (B) Sequence alignment of similar actin binding domains from other human proteins. Residues at the 18^th^, 39^th^, and 41^st^ positions are shown in red, green, and blue colors respectively. (C) Structural view of stabilizing interactions formed by the sidechain of K18 in the WT dystrophin structure. All atoms that come close to the amide nitrogen of the lysine sidechain are shown by connecting green lines.

Sequence and structural analysis suggest why K18N mutation can be destabilizing (Fig. 5). The lysine residue at the 18^th^ position is highly conserved in mammals (Fig. 5A) and in similar functional domains from other human proteins (Fig. 5B), which implies that this residue is essential for protein structure and/or function. This residue is part of the first helix in dystrophin (Fig. 1A). It stabilizes the turn connecting the first and second helices by forming a salt bridge with the sidechain of asparagine at position 39 and by forming a cation-pi interaction with the aromatic sidechain of phenylalanine at position 41 (Fig. 5C). Both residues 39 and 41 are also highly conserved in mammals (Fig. 5A) and across similar functional domains (Fig. 5B). Whenever there exists a lysine at the 18^th^ position, the residues at positions 39 and 41 are always asparagine and an amino acid with a phenyl ring (either phenylalanine or tyrosine) respectively. In addition, the K18 sidechain also forms a stabilizing hydrogen bond with a bound water molecule (Fig. 5C). Therefore, mutating this residue is expected to break these various stabilizing interactions, which will destabilize the dystrophin structure.

A similar situation occurs in other diseases where decrease in protein stability appears to be the major triggering factor. In cystic fibrosis, the ΔF508 mutation in cystic fibrosis transmembrane regulator (CFTR) protein, which occurs in 70% of cystic fibrosis patients, has a decreased stability compared to WT, leading to a decrease in the functional protein concentration *in vivo* and hence net decreased function [37], [38]. Carcinogenic mutations in the tumor suppressor protein p53 reduce the protein stability. Folded p53 levels *in vivo* were correlated with changes in protein stability [39], [40]. Disease-causing mutations in rhodopsin decrease its thermodynamic stability [41]. Although missense mutations can trigger disease by multiple routes, decrease in stability seems to be the major factor that is responsible for ∼80% of monogenic missense mutation-triggered diseases in proteins [42].

It is interesting to compare the K18N mutation with other dystrophin mutations that trigger Duchenne/Becker muscular dystrophy (DMD/BMD). Similar to K18N, DMD/BMD mutations also decrease protein stability, which may account for decreased dystrophin levels observed in DMD/BMD patients [18], [43], [44]. However, the major difference between K18N and DMD/BMD mutations is protein aggregation. DMD/BMD mutants undergo severe protein aggregation, whereas K18N does not aggregate. Whether aggregation plays a major role in DMD/BMD and not in XLDCM is not clear. In addition, why K18N has no effect on skeletal muscles is unclear. These aspects need to be further examined.

One caveat in this study is that we are studying isolated N-ABD instead of the full-length human dystrophin. High-yield expression and purification of full-length human dystrophin to acceptable purity is not yet possible. Further, full-length dystrophin is very large in size (∼427 kDa), and is not amenable to many high-resolution structural and biophysical techniques. Therefore, we follow a reductionist approach of studying individual domains, similar to studies on other dystrophin domains that include N-ABDs [18], [28], [33], [34], [45], spectrin repeats [46]–[52], and C-terminal domains [53]. Because the full-length human protein is not yet possible to express and purify, effects of disease-causing mutations on the structure and function of dystrophin are in general probed at the level of individual domains [18], [28], [50], [51], [54]. In addition, determining the mutation effects on full-length dystrophin solution structure on the scale of individual amino acids (Fig. 3) is not possible with the available structural methods. Typical protein size for which solution NMR methods can be currently applied needs to be less than 300 amino acids.

Our results on human dystrophin N-ABD agree to some extent with an earlier study on full-length mouse dystrophin [44]. In this earlier work, which involved the expression and purification of full-length mouse dystrophin, the K18N mutant showed an α-helical circular dichroism as that of the WT dystrophin [44], similar to that observed here for human dystrophin N-ABD (Fig. 1C). However, the mutation resulted in the loss of sigmoidal transition for mouse dystrophin during its temperature melt [44], in contrary to the sigmoidal melt observed here for the K18N mutant of human dystrophin N-ABD (Fig. 2B). Further, the K18N mutation resulted in a significant aggregation of mouse dystrophin [44], whereas the human dystrophin N-ABD mutant did not aggregate (Fig. 1F). These differences can be because of two possible reasons. The first reason is that we are studying the mutation effects on isolated N-ABD rather than the full-length protein. The second possible reason is that human dystrophin may differ from mouse dystrophin in terms of protein structure and function. These aspects need to be further probed.
